# Sharing Employee: B2B Employment Model in the Era of Coronavirus Disease 2019 and Implication for Human Resource Management

**DOI:** 10.3389/fpsyg.2021.714704

**Published:** 2021-09-09

**Authors:** Zhisheng Chen

**Affiliations:** College of Economics and Management, Nanjing University of Aeronautics and Astronautics, Nanjing, China

**Keywords:** B2B, COVID-19, human resource management, Sharing Employees, employment

## Abstract

With the explosion of coronavirus disease 2019 (COVID-19), the concept of “Sharing Employees” has emerged in China. This study will discuss the background of the formation of the “Sharing Employees,” how the “Sharing Employees” model is implemented, the relative risks, and the impact on human resource management. Currently, this virus is spreading worldwide, affecting the economy and increasing the unemployment rate. This study will help other countries to learn from this model and provide suggestions for adopting flexible employment policies to ease employment pressure and increase employment channels through the “Sharing Employees” B2B model.

## Introduction

The number of sharing economy business models has increased significantly in recent times. The rapid development of the sharing economy and its huge impact on different aspects of the current socio-economic system has stimulated public interest (Cheng, 2016). In the era of coronavirus disease 2019 (COVID-19), it is becoming more and more important to promote more sustainable and more promising sharing forms and use their benefits while avoiding traps (Mont et al., 2020). The form creates sustainable value, that is, increasing social welfare, lessening environmental load, and providing economic benefits, for example, avoiding excessive consumption, allowing more efficient and sustainable use of underutilized resources, establishing deeper social connections between people and changing consumption habits (Laukkanen and Tura, 2020).

With the impact of COVID-19 in China, the concept of “Sharing Employees” came into being. This concept is somewhat like the sharing economy, but what it shares is the labor forces. COVID-19 has caused tremendous damage to work and workers, especially those who are unemployed (Fouad, 2020). Under the ongoing impact of the epidemic, some small and medium-size enterprise (SMEs) that were temporarily unable to restart their businesses dispatched their excess labor to other companies in need of employees on a shared basis. The cooperative employment model effectively alleviated the shortage of temporary workers for companies whose business volume increased during the epidemic.

This study introduces the “Sharing Employees” approach as a B2B model. It differs from the traditional employment approach and therefore poses some challenges to human resource management: how to confirm the labor relationship, how to pay wages, who pays social insurance, and who conducts professional training.

This study proposes a solution to the “Sharing Employees” problem. The employee relationship belongs to the dispatching enterprise and will not be unclear because of their task assignment. Based on the issue of wage payment, the dispatching enterprise is responsible for paying the basic wage and the “borrowing” enterprise is paid according to the workload; social insurance is borne by the dispatching enterprise; after the shared employees are dispatched, the “borrowing” enterprise is obliged to provide corresponding vocational skills and safety training. This study clarifies the corresponding “shared employee” issue, which helps to delineate the rights and responsibilities of both parties.

In terms of future development, this model can, on the one hand, solve the problem of redundant labor outlets, stabilize the workforce, and avoid layoffs during special periods; on the other hand, it can solve the problem of labor shortage for new retail enterprises, ensure the orderly supply of the market, and win public praise; furthermore, it can build a cross-industry temporary employment ecology under the pandemic (Qing, 2020).

## Literature Backgrounds

For most of the past century, work is usually described as a regular, full-time employment mode defined as work performed on a fixed schedule, at the business location of the company under the control of the company, expecting to continue to hire (Spreitzer et al., 2017). In the process of traditional economic development, people are more inclined to formal employment, and the employment relationship exists between enterprises and workers generally. Some scholars put forward that flexible employment is different from fixed full-time employment, which means that enterprises can flexibly hire people according to their respective needs, and the enterprise and the employee do not establish a formal full-time labor relationship. Generally, flexible contracts also have no impact on the overall life satisfaction of the employed (Green and Heywood, 2011). However, the flexibility of the B2B employment model is not embodied in both employees and employers in the same company, but in the transfer of labor from companies with redundant labor to companies with insufficient labor.

According to Spreitzer, the flexibility of work arrangements can be categorized within three dimensions as follows: (a) flexibility in the scheduling of work, (b) flexibility in the location where work is accomplished, and (c) flexibility in the employment relationships (Berber and Slavić, 1999). In the B2B model, the employment relationship is not fixed. During the special period of COVID-19, employees can move between different companies and join them at different times according to their respective labor needs. Flexible employment models include labor dispatch (Purcell and John, 1998), business outsourcing (Purcell and John, 1998), reemployment of retirees, short-term labor contract workers, part-time workers, etc. The recently emerged B2B employment model is an innovative flexible employment method that is different from the previous ones.

The term “sharing economy” first appeared in 2008 and refers to “collaborative consumption” resulting from the activities of exchanging, sharing, and leasing resources without owning commodities (Petrini et al., 2017). In economic transactions, it refers to the use of good physical goods or services whose consumption is broken down into individual parts (Puschmann and Alt, 2016). Consumers always benefit from collaborative consumption (Benjaafar et al., 2019). The “Sharing Employees” model essentially refers to the sharing of labor across different businesses, as it is also an evolution of the “sharing economy” concept. It reflects the exchange of labor between one B (business) and another B (business). In general, companies can hire and share labor flexibly through the B2B employment model, so it is not difficult to find that this model absorbs the advantages of “flexible employment” and “sharing economy.”

The essence of B2B is also a kind of flexible employment in the form of “odd job” in China (Daming, 1997). In the 1980s, township enterprises of China began to flourish and faced a shortage of skilled and productive professionals. As a result, township governments and enterprises rehired retired technicians and engineers on Sundays to help the enterprises. This was helpful in achieving an on-demand supply of technical and human resources during the special period. They were referred to as “Sunday Engineers” (Journal, 2020). In fact, this form of “odd job” is common at present. The subtle difference is that the “Sharing Employees” at the time of the COVID-19 crisis was a collective lease relationship established by two companies, not an individual and a company.

Flexible work is also used in international labor standards. Not only has it created a clear employment miracle since the economic crisis but also according to the Organization for Economic Cooperation and Development (OECD), it also provides a model for other European countries to follow (Rubery et al., 2016). Many Internet companies do not require their employees to clock in every day and do not have mandatory working hours. Some people with the appropriate professional skills can also work from home. Many companies need to hire an “odd job” by flexible employment policy during travel and peak seasons of the hotel (Krakover, 2000). In this sense, the concept of the “Sharing Employees” is closer to the “Gig Economy,” but the process of the “Sharing Employees” is influenced by both companies rather than individuals.

In general, compared with the traditional employment relationship, the “Sharing Employees” model has the following advantages: first, the sharing of employees has greatly changed the traditional way of employment. The relationship between enterprises and employees is not only the employment relationship but also the relationship of sharing and cooperation; second, “Sharing Employees” solves the problems in the special epidemic period when allocating the labor forces among different enterprises and realizing the reasonable flow and optimal allocation of the labor force; third, the “Sharing Employees” mode avoids the intermediary mode in which the third-party human resource companies allocate the labor demand among enterprises, reduces the labor cost, invigorates the human capital of the enterprise, and enhances the manpower flexibility.

## The Forming Practical Background of “Sharing Employees”

At the beginning of 2020, due to the continuing impact of COVID-19, some Chinese enterprises that are temporarily unable to renew business are under great pressure to pay basic salaries for their employees (Liu et al., 2020). Companies affected by the epidemic have raised the level of innovation to survive (Wang et al., 2020). At the epidemic time, because of the surging demand for online shopping, there are plenty of vacancies for the position of stores staff and deliverers in online retail enterprises, hence the emergence of the new employment model of “Sharing Employees.” From the perspective of the background of “Sharing Employees,” it refers to the reasonable allocation of labor resources in different enterprises according to their needs in such a special economic environment of COVID-19, so as to achieve a win-win employment model among manpower dispatching enterprises, manpower “borrowing” enterprises.

The “Sharing Employees” model provides the job chance of more than 4 million catering workers in China. On January 23, 2020, under the influence of COVID-19, Wuhan city of China announced “Closure of the City,” and then all provinces and cities in China successively initiated the first-level response to a major public health emergency (Zhao, 2020). Restaurants, hotels, entertainment, department stores, malls, and other business shut down within a few days. On February 3, Hema Xiansheng supermarket announced that it would accept some employees of Yunhaiyao Catering Company to work in the Hema store (Xi, 2020) and admit redundant employees from various industries. These employees will attend induction training and can only work after they pass the training. The decision of Hema was the prelude to a “Sharing Employees” business model in China.

With the online retail industry offering an olive branch to “Sharing Employees” in China, the “Sharing Employees” model has been producing new breakthroughs in various industries, gradually spreading from online retail industry to logistics, manufacturing, and other industries and expanding from Chinese first-tier cities to second- and third-tier cities. On February 8, Lenovo Group provided job positions related to computers, servers, and mobile phone assembling tasks, in an attempt to help small- and medium-sized enterprises survive the “winter” through the “Sharing Employees” model and solve the insufficient manpower in various Lenovo factories (Li, 2020).

The current new coronary pneumonia has caused serious economic consequences globally, and it seems that any country will be affected, which has led to huge changes in business and consumer behavior (Donthu and Gustafsson, 2020). Many developed countries are facing two-digit unemployment rates forcing people to seek unemployment benefits (Bong et al., 2020). As it reaches the low- and middle-income countries, its effects could be even more dire. It is estimated that the reduction in working hours by April 1, 2020 is equivalent to a reduction of 200 million full-time jobs in Europe (Yamin, 2020). The International Labor Organization estimates that in the second quarter of 2020, due to COVID-19, employment has fallen by 10.5%, which means the loss of 309 million full-time jobs (Ceylan et al., 2020).

Fighting a global pandemic requires large-scale cooperation. The problem is that, by definition, collaboration requires people to bear personal costs to benefit others. There is a conflict between short-term self-interest and long-term collective interests. In addition, in this epidemic, there are many groups (for example, family, community, national, and international) that can make cooperative decisions. From an evolutionary viewpoint, expanding self-interest to maintain and promote the welfare of family members should be a small step, because it improves the adaptability of genes. Therefore, a major issue is how to promote cooperation (Van Bavel et al., 2020). In fact, the epidemic has hit western economies with shops closing and more people facing unemployment. Therefore, it is necessary to strengthen the innovative cooperation between different enterprises at a special time. It is highly recommended to refer to the “Sharing Employees” method to relieve the unemployment pressure caused by the impact of COVID-19. On the one hand, this model can help enterprises with a large number of idle employees to relieve cost pressure and ensure cash flow; on the other hand, it can help some enterprises solve the “labor shortage” problem due to the sudden boom of business in the time of COVID-19 (Qian, 2020).

## “Sharing Employees”: Model, Method, and Risk

**Figure 1:**
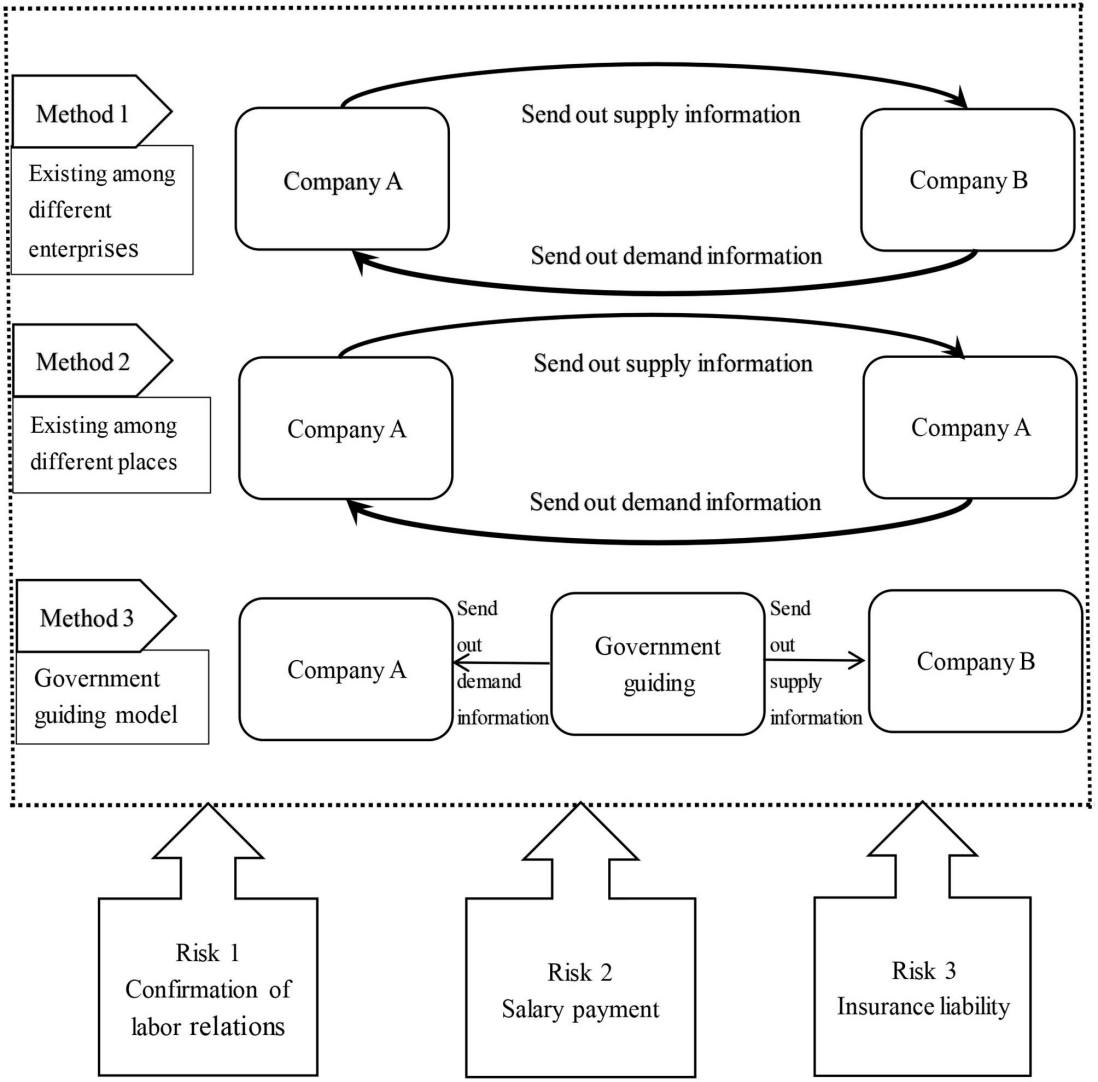
“Sharing employees” model, method, and risk.

In special times, companies use the employee sharing approach. First, we must analyze the characteristics of the model; second, what methods are used to implement the model; and, in addition, the legal risks associated with the model. Figure 1 shows the “Sharing Employees” model, methods, and risks.

### B2B Model of “Sharing Employees”

The current “Sharing Employees” model on the market is a B2B format, where both the supplier and the recipient of the employee implement a share agreement. This model reflects a direct link between the two organizations. One party oversees the export of labor and the other is responsible for the import of labor. Both parties sign a secondment contract. Of course, they will face a lot of legal issues, such as the confirmation of labor relations, wages, and the responsible party after a work injury.

The first is the demand side, which can be a company or an organization. It can only be a demand party if it has a need and is willing to accept surplus employees from other organizations. After the outbreak, in many industries affected by COVID-19, such as take-out, supermarkets, and travel across the country, some employees could not return to work on time due to the prevention policy. However, many companies, such as Haima Xiangsheng and Suning Tesco, urgently needed more employees because of the surge in business volume. However, it is difficult to recruit suitable workers in the short term. Dozens of “Sharing Employees” demand programs of companies were launched one after another, becoming the main demand side.

The second is the supply side, which can also be a company or an organization; after COVID-19, employees who are not on assignment stay at home and need to be paid by the company. To reduce labor costs, these companies are eager to join “Sharing Employees” programs, and employees are eager to join to increase their income. Companies with many redundant employees post information on labor supply, including the number of available employees, basic skills, suitable positions, and available hours. Through the exchange of information between the demand side and the supply side, the B2B model is established.

### Three Ways to Realize the “Sharing Employees” Model

First, the “Sharing Employees” model exists among different companies. Haier Industrial Park in Hefei, China, an economic development zone, attracts 119 shared employees from other companies (58 from Anhui Century Jinyuan Hotel, 25 from Chotai Security, and 36 from Anhui Jinling Hotel). Jingdong Logistics of China has also partnered with companies with redundant employees, such as Kawanka and Zhibang Kitchen Cabinet, and has taken on 24 shared employees from these companies.

Second, the “Sharing Employees” model exists in different work locations of the company. For example, Anhui Meiling Company in China had an increase in overseas orders and needed labor for its rapid production demand, but due to the pandemic, there were not enough employees returning. With the support of the local government, Anhui Meiling transferred 598 employees from Sichuan Meiling and 157 employees from Jiangxi Meiling through an internal “Sharing Employees” model. These measures realized the temporary mobility of shared employees in different workplaces within the group.

Third, the government participated in guiding the B2B program. The public administration of the government acted as an intermediary coordinator for the transfer of shared employees between different companies.

### Legal Risks of the B2B Model

Confirmation of labor relations. The labor exporting company and its employees still maintain labor relationships, and the labor exporting company and the labor importing company do not need to obtain or pay labor dispatch fees under the B2B model. If the exporting company forcibly requires the employee to participate in the B2B plan, the employee has the right to terminate the labor contract and claim financial compensation; if the labor exporting company terminates the labor contract on the grounds that the employee does not agree to the “Sharing Employees” plan, it is considered as an illegal termination of the labor contract and should compensate the dispatched employee.

Wage payment. Under the B2B model, the main body of wage payment is established according to the agreement between the labor exporting company and the labor importing company. According to the legal secondment relationship, the labor importing enterprise generally pays wages to the labor exporting enterprise first, and the labor exporting enterprise then pays wages to the shared employees. Neither party may withhold wages belonging to the shared employee.

Identification of work-related injury insurance liability. Under the B2B model, the labor exporting company should bear the responsibility of work injury insurance because the labor relationship still belongs to the labor exporting company. After the work injury occurs, the labor exporting company is obliged to assist the employee to apply for work injury insurance treatment.

## Implication on Human Resource Management

At present, the researcher article is of interest to our current reincarnation as a “strategic partner” in organizations today. Sikora and Ferris proposed that the real problem facing strategic human resource management (HRM) is human resource (HR) implementation (Deadrick and Stone, 2014). The Human Resource Department acts as a strategic partner, focusing on the business operations of the entire company and changes in the external environment. In the current social context, the spread of the new coronavirus has severely impacted the business of many companies. Some companies are understaffed, while others are overstaffed. Facing the changes in the external environment, HRM should make strategic arrangements and as a key role help their labor force cope with and adjust to their newly altered work environment (Carnevale and Hatak, 2020).

This study will further consider the alignment of HR functions, such as HR planning, recruitment, compensation, and employee relations, based on the B2B employment model. In the B2B model, employees are hired on a temporary basis. Shared employees experience some internal insecurities, such as feeling insecure about their employment, pessimistic about the future arrangement, and worrying about their lack of compensation insurance and pension benefits of workers (Feldman et al., 1994). Therefore, there is a need to regulate human resource management in both labor exporting and labor importing firms and to implement the B2B model. Tables 1, 2 provide a description of supply-side and demand-side HRM.

**Table 1:** The description of supply-side HRM.

Human resources planning	It is necessary to make advance human resource planning for redundant employees and contact companies with labor demand by the “Sharing Employees” method.
Salary and performance	Shared employees' wages during the “borrowing” period are borne by the “borrowing” company and paid by the dispatching company. They should be assessed although dispatched employees work mainly in the labor importing company.
Employment relationships	The two companies form a “Shared Employees” cooperation model through secondment method, and must sign a secondment agreement. Labor exporting enterprises should continuously purchase industrial injury insurance for shared employees.
Employee Psychological Counseling	The dispatching company should give psychological comfort to the shared employees. They can still return to the dispatching company after completing the work of borrowing company.
Training and career planning	The dispatching company to provide the previous training list, which can be used as a reference for the “borrowing” company. The acquiring multiple skills is also valuable to the career development of shared employees.
Establishment of sharing information platform	Enterprises with redundant employees deliver information, which can include shared employee's skills, age, and dispatching time
Communication between Dispatching and “Borrowing” Enterprises	The dispatching company should proactively pass past job performance of the shared employees to the “borrowing” company.

**Table 2:** The description of demand-side HR.

Human resources planning	Human resource demand should be planned, and actively seeking to contact companies with redundant employees to meet short-term business needs.
Salary and performance	It is necessary to inform of salary standards. Shared employees' wages during the “borrowing” period are borne by the “borrowing” company and paid by the dispatching company. A judicious assessment of the workforce value is useful.
Employment relationships	Labor importing enterprises purchase commercial insurance and inform them of industry characteristics and job requirements.
Employee psychological counseling	Workforce “borrowing” companies should assuage employee concerns of reasonable wages, fairness in performance assessment, on-job training, and labor security.
Training and career planning	It is necessary to deliver the relative training for shared employees to help familiarize themselves with the new workplace and job positions. Employees of the B2B model have acquired the skills of the dispatching company, and gained new job skills from the “borrowing” company.
Establishment of sharing information platform	“Borrowing” enterprises also actively searches for information about available shared employees by the on-line platform.
Communication between Dispatching and “Borrowing” Enterprises	The “borrowing” company should communicate with the dispatching company about their cultural adaptability, further considering whether to need some workforce reserves.

### Human Resource Planning

Facing the epidemic situation, the uncertain factors of the future will increase, and human resource planning should be adjusted reasonably. Affected by COVID-19, the business of the company declined and cost-efficiency decreased. Cost-effective strategies tend to reduce production costs and achieve high-capacity utilization (Abdul-Halim et al., 2016). Based on the degree of the epidemic influence and the business decline, the human resource plan should be adjusted and implemented in stages. In the initial stage of COVID-19, epidemic damage was not severe and employees could work at full capacity. It is unnecessary to adjust the human resource management plan because there is no redundant or lack of workforce. As the impact of the epidemic has deepened, some countries, including the largest economy in the world, have required most of the businesses to close, limiting the opportunities for people to gather and move. These preventive actions have a direct and significant impact on both domestic and international companies (Liu et al., 2020). Business shrinkage leads to employee unemployment or reduces working hours, and at the same time, the business of some companies is soaring and urgently needs employees. It is necessary to make advance human resource planning for redundant employees and contact companies with labor demand by the “Sharing Employees” method.

Have a look at the demanding body of the human resources of the model. Surprisingly, because of the impact of the epidemic, the business of some companies has skyrocketed, requiring more employees but unable to recruit large amounts of labor in the short term. Human resource demand should be planned, actively seeking to contact companies with redundant employees to meet short-term business needs. On the one hand, it eases the pressure on short-term workforce shortage; on the other hand, the labor cost of dispatched enterprises is greatly reduced by the B2B method. Therefore, proper human resource planning will achieve a solution where everyone benefits.

### Salary and Performance

The efforts of workers depend on their perception of whether they are treated fairly in an efficient wage model (Palley, 1994). Salary satisfaction and salary fairness are the most concerns of employees and employers (Suleiman, 2014). According to the “Sharing Employees” model, dispatched employees do not work in the dispatching company, and their income and benefits should be paid by the labor importing company. However, there is a case that working sometimes in the dispatching company or in the labor importing company. Both companies should calculate their income based on the labor amount. Both companies are obligated to make a reasonable explanation for their employees: the payment method and the calculation method of salary. About the job performance, if part of the working hours is in the dispatching company, the agreeing performance evaluation should also be calculated by the dispatching company. In daily performance evaluation, they should be fairly assessed although dispatched employees work mainly in the labor importing company.

As a labor force importing company, it is necessary to clarify salary standards and performance measurement methods when conducting induction training and stipulating contracts for dispatched employees. Although the labor forces exporting company and importing company belong to different industries, and the salary standards will also be different, it is necessary to inform them in advance. If not, it will cause a deviation from understanding salary policy and possibly form a new employment conflict. A judicious assessment of the workforce value is useful to cooperate better between both companies in the future.

### Employment Relationships

In the B2B model, the two companies form a “Shared Employees” cooperation model through the secondment method and must sign a secondment agreement. It is possible that shared employees not only gain lower salaries and benefits than regular employees but also lack mature legal protection for labor relations. At the same time, there are also many disadvantages of recruitment, assessment, rewards, and punishments. A clear employment contract can reduce the factors of inequality, but we should also note that many employees judge the employment relationship through the perceived informal psychological contract rather than external regulatory obligations (Atkinson et al., 2016). Trust plays a central role, and it provides useful insights into modern employment relations.

Both parties must agree on their respective rights, duties, and responsibilities and explain job responsibilities, working time, salary calculation methods, safeguards, legal risks, and liability attribution. For example, labor accidents could occur during the “manpower lending.” Labor exporting enterprises should continuously purchase industrial injury insurance for shared employees. At the same time, labor importing enterprises purchase commercial insurance, which is a useful supplement to work injury insurance. Enterprises with short-term workforce demand should present industry characteristics and job requirements to guarantee that the “borrowing” workers can effectively and quickly be competent for vacant positions.

### Employee Psychological Counseling

Labor rights activists worry that odd jobs are associated with increased risks of workers, including unstable and demanding employment conditions (Spurk and Straub, 2020). Employees participating in the B2B program share the same concerns that once they participate in the “Sharing Employees” plan, they possibly lose the dispatching position of the company and are unable to adapt to the new position of the “borrowing” company. The dispatching company should give psychological comfort to the shared employees: according to the dispatching agreement, the dispatching plan is temporary rather than permanent, and eventually, they will return to the dispatching company. Workforce “borrowing” companies should assuage employee concerns of reasonable wages, fairness in performance assessment, on-job training, and labor security.

Short working hours can easily lead to these unsuitable working attitudes and low-work efficiency of employees, so influencing normal production. Because of social and family pressure, someone may often choose a long-term and stable job. When the epidemic is over, the enthusiasm of “Sharing Employees” is likely to be diminished (Mengdi, 2020). Furthermore, human resource management may make psychological counseling and health tracking plan to prevent psychological discomfort of employees and negative impact on their work when the shared employee is in service.

### Training and Career Planning

It is necessary to deliver the relative training for shared employees to help familiarize themselves with the new workplace and job positions, understand the basic working procedures and work requirements, and operate necessary protection facilities. Staff whose line managers spent time discussing career plans, development opportunities, and training needs felt more engaged, listened to, and valued (Holmes, 2020). Since they have a different understanding of new positions and job procedures, they need to spend a lot of time familiarizing themselves with job responsibilities and products. Fitting training helps them quickly understand the business; otherwise, the operational flexibility should be influenced, and the duties of the position cannot be fulfilled quickly. It is also indispensable for the dispatching company to provide the previous training list, which can be used as a reference for the “borrowing” company, reduce the unnecessary training cost, and increase the training efficiency. Overall, training improves the qualifications, skills, and career development of employees, so it can have a positive impact on shared employees and organizations (Ma et al., 2020).

The COVID-19 epidemic is a career shock for many people across the world (Akkermans et al., 2020). The change of micro and macro boundaries is one of the important mechanisms of COVID-19 affecting individual professional behavior and career outcomes (Cho, 2020). Such training is also comparably valuable for shared employees, who gain additional experience to help pursue their chosen career paths. Workers need to adapt their careers to ever-changing demands and circumstances, a possible challenge for employees of traditionally stable organizations (Der Horst and Klehe, 2019). Employees of the B2B model have acquired the skills of the dispatching company and gained new job skills from the “borrowing” company. They can adapt to the previous company as well as the current company. It should be stated that acquiring multiple skills is also valuable to the career development of shared employees.

### Establishment of Sharing Information Platform

“Shared employees” provide a new way to resolve the labor imbalance. Companies whose employees are in short supply cannot exchange workforce with other companies of surplus employees because the information is asymmetric. The peer-to-peer-based activity of sharing the access to coordinated services has been expected to alleviate societal problems (Hamari et al., 2016). The peer-to-peer platform could become an available tool for the purpose of workforce information sharing. The establishment of an information platform for shared employees should generally include the following three factors: idle resources, shared network platforms, and many participants, all of which are indispensable. A mature and perfect platform could match timely, fastly, and accurately workforce supply and demand parties with the help of digital technology and Internet big data.

Once online information platforms are developed well, it is helpful for both companies to use the platform to communicating and sharing employee information. The information platform can be built between enterprises or led by governments and institutions. The purpose of building an information platform is to exchange labor information. Enterprises with redundant employees deliver information, which can include shared skills, age, and dispatching time of employees; companies lacking employees can also post messages, which include the number of people in need, the required skills, salary, and “borrowing” time. Of course, “borrowing” enterprises also actively search for information about available shared employees by the online platform. The “Sharing Employees” platform also needs to establish a credit evaluation mechanism and open the credit scores of enterprises participating in the sharing plan. The mechanism may provide a reference basis for the cooperation between both parties.

### Communication Between Dispatching and “Borrowing” Enterprises

There may be cultural in-adaptability of shared employees in the “borrowing” enterprises, which will result in low stability and high management costs. The “borrowing” company should communicate with the dispatching company about their cultural adaptability, further considering whether to need some workforce reserves.

In the traditional work model, performance evaluation is conducted in terms of moral character, knowledge, and ability, but in the “shared” model, the work is short-term and temporary. It is difficult to make an effective evaluation on shared employees, even if they complete the task as scheduled. It is suggested that the “borrowing” company should obtain past job performance of the shared employees from the dispatching company before starting employment.

Both employers need to clarify the rights and obligations in the secondment agreement. It is also required that both parties agree with the salary and payment standards of shared employees to avoid disputes. The “borrowing” enterprise arranges suitable positions according to the skills and job requirements of employees. Wages and benefits of shared employees are determined by working hours or the number of products. The wages of shared employees during the “borrowing” period are borne by the “borrowing” company and paid by the dispatching company.

The “Sharing Employees” model will make the relationship between enterprises and employees more complex, and multilevel labor relations will coexist. The existing labor laws and regulations are obviously insufficient for new-type labor relations and have weak control over possible complex disputes, especially those involving business secrets. If the labor dispute with the “borrowing” company is caused by leakage of business secrets, the “borrowing” company should also promptly give dispatching company feedback and seek a solution (Li, 2020).

## Prospects for the B2B Employment Model

Starting with the COVID-19 epidemic, the “Sharing Employees” model will persist as the sharing economy evolves. This model contributes to the efficient use of labor and facilitates labor mobility between surplus and deficient labor companies. As the impact of COVID-19 deepens, it will permeate industries, regions, and countries. Currently, COVID-19 is spreading worldwide, with many employees facing unemployment while some industries are in desperate need of employees. This model has a clear effect on countries around the world to better utilize their workforce during the COVID-19 impact. It is foreseeable that the future society is a sharing economy society, and only resource-sharing can promote the maximum benefit of all parties. The labor force is the most valuable resource for enterprises. Labor, as one of the factors of production, can only be maximized by the most effective flow between enterprises. In the future, more people will face the world with flexible and free working conditions.

There is still a long way to go in the development of the B2B model, which may be adapted with a third platform (Mingliang, 2020). Some scholars point out that the B2B model can only be used as a contingency measure if it is only an inter-company labor allocation as being utilized in special periods. Overall, the “Sharing Employees” model has a stronger vitality by integrating social resources, enterprise resources, and employee resources through the online App. Once a networked platform is established, it will accelerate the more effective integration of labor resources. How to establish a third-party network-sharing platform dedicated to serving diversified enterprises and employees will become a new issue.

## Limitations of the Research and Future Research Directions

The idea of a B2B model of shared employees emerged with COVID-19, so there is not much literature on “Sharing Employees.” This also leads to an inadequate exploration of the literature for this study. However, the authors have analyzed the B2B employment model from the sharing economy and flexible work arrangement literature. This study focuses on qualitative analysis, and quantitative analysis on this model is lacking. Due to the pandemic, business development has been affected, and it is difficult to collect and study the underlying business data.

Future research on the B2B employment model is needed. This research on the “Sharing Employees” model is applicable in the current COVID-19 crisis, but in the post-COVID-19 era, there is a need to examine how the model can continue to develop and function in the enterprise. In addition, this model is considered as an inspiration for HRM, and we need to further consider the psychological changes and responses of employees under this model.

## Conclusion

While the COVID-19 pandemic is affecting economies around the world, the “Sharing Employees” model, which uses flexible employment methods, has eased social production difficulties in special times, and surplus labor has been placed in different industries. The “Sharing Employees” model, as a new and innovative way of employment in the sharing economy, has become a hot topic of social research. The three ways of shared employees effectively help enterprises to use shared labor flexibly, but the corresponding legal risks should not be ignored. The emergence of the sharing model has put forward new requirements for dispatching and “borrowing” companies, including human resource management planning, employee relations, pay and performance, information platforms, psychological counseling, and career planning. In the future, this model will be a long-term one, and it is a trend to realize the “Sharing Employees” model through commercial network platforms.

## Author Contributions

ZC contributed to conception and design of the study. He also contributed to manuscript revision, read, and approved the submitted version.

## Conflict of Interest

The author declares that the research was conducted in the absence of any commercial or financial relationships that could be construed as a potential conflict of interest.

## Publisher's Note

All claims expressed in this article are solely those of the authors and do not necessarily represent those of their affiliated organizations, or those of the publisher, the editors and the reviewers. Any product that may be evaluated in this article, or claim that may be made by its manufacturer, is not guaranteed or endorsed by the publisher.
